# Treatment results for severe psychiatric illness: which method is best suited to denote the outcome of mental health care?

**DOI:** 10.1186/s12888-018-1798-4

**Published:** 2018-07-13

**Authors:** Edwin de Beurs, Matthijs Blankers, Philippe Delespaul, Erik van Duijn, Niels Mulder, Annet Nugter, Wilma Swildens, Bea G. Tiemens, Jan Theunissen, Arno F. A. van Voorst, Jaap van Weeghel

**Affiliations:** 1 Stichting Benchmark GGZ, Rembrandtlaan 46, 3723 BK Bilthoven, Netherlands; 2 Leiden University, Wassenaarseweg 52, 2333 Leiden, AK Netherlands; 3 Arkin, Klaprozenweg 111, 1033 Amsterdam, NN Netherlands; 4 Trimbos Institute, Da Costakade 45, 3521 Utrecht, VS Netherlands; 5 Maastricht University, Minderbroedersberg 4-6, 6211 Maastricht, LK Netherlands; 6 GGZ Delfland, Sint Jorisweg 2, 2612 Delft, GA Netherlands; 7 Parnassia Bavo GGZ Zorgholding BV, Monsterseweg 93, 2553 Den Haag, RJ Netherlands; 8 Erasmus University, Burgemeester Oudlaan 50, 3062 Rotterdam, PA Netherlands; 9 GGZ Noord-Holland Noord, Postbus 18, 1850 Heilo, BA Netherlands; 10 Altrecht Mental Health Care, Lange Nieuwstraat 119, 3512 Utrecht, PG Netherlands; 11 Pro Persona Mental health Services ProCES, Indigo, Wolfheze 2, 6874 Wolfheze, BE Netherlands; 12 Radboud University, Comeniuslaan 4, 6525 Nijmegen, HP Netherlands; 13 GGZ Ingeest, VU University Medical Center Amsterdam, De Boelelaan 1117, 1081 Amsterdam, HV Netherlands; 14 GGZ Centraal, Utrechtseweg 266, 3818 Amersfoort, EW Netherlands; 15 Phrenos, Trimbos Institute, Da Costakade 45, 3521 Utrecht, VS Netherlands; 16 Tilburg University, Warandelaan 2, 5037 Tilburg, AB Netherlands

**Keywords:** Clinical significance, HoNOS, Routine outcome monitoring, Severe mental illness, Treatment outcome

## Abstract

**Background:**

The present study investigates the suitability of various treatment outcome indicators to evaluate performance of mental health institutions that provide care to patients with severe mental illness. Several categorical approaches are compared to a reference indicator (continuous outcome) using pretest-posttest data of the Health of Nation Outcome Scales (HoNOS).

**Methods:**

Data from 10 institutions and 3189 patients were used, comprising outcomes of the first year of treatment by teams providing long-term care.

**Results:**

Findings revealed differences between continuous indicators (standardized pre-post difference score ES and ΔT) and categorical indicators (SEM, JT_RCI_, JT_CS_, JT_RCI&CS_, JT_revised_) on their ranking of institutions, as well as substantial differences among categorical indicators; the outcome according to the traditional JT approach was most concordant with the continuous outcome indicators.

**Conclusions:**

For research comparing group averages, a continuous outcome indicator such as ES or ΔT is preferred, as this best preserves information from the original variable. Categorical outcomes can be used to illustrate what is accomplished in clinical terms. For categorical outcome, the classical Jacobson-Truax approach is preferred over the more complex method of Parabiaghi et al. with eight outcome categories. The latter may be valuable in clinical practice as it allows for a more detailed characterization of individual patients.

## Background

Routine Outcome Monitoring (ROM) is gathering momentum as an adjunct to treatment [1, 2] and as a basis for outcome management [3]. In the Netherlands, ROM has been stimulated by health insurers, resulting in a nationwide implementation of ROM in clinical practice to serve both goals: providing feedback on individual treatment progress and on outcomes attained with groups of patients (aggregated outcomes). The present paper focuses on the latter. Currently about 45% of all remunerated treatments can be evaluated, and results are aggregated and used to give feedback to institutions on their performance in terms of outcome [4]. For ROM assessments of patients with severe mental illness (SMI), the Health of Nation Outcome Scales (HoNOS) [5] is used, a well-known rating scale generally completed by the professional who delivers care. The HoNOS comprises 12 items, each with five response options (scoring range is 0–48), and has good psychometric properties [6]. Outcome on clinical problems and psychosocial functioning is assessed by comparing pretest and posttest total scores on the HoNOS for each patient. The simplest, most straightforward and most commonly used outcome indicator in treatment outcome research is the average change from pretest to posttest score, converted into a standardized change score or within- group effect size (ES) indicator [7, 8]. For benchmarking in the Netherlands, we have adapted this approach to a change score based on transformed T-scores (ΔT) [9]. However, average change offers rather abstract information on the performance of treatment institutions. It would be informative to know what proportion of patients have benefitted from treatment or can be considered as recovered, yielding a performance indicator with direct appeal.

Jacobson et al. [10–12] have proposed a method to delineate the treatment results of individual patients, comprising criteria for clinically significant and statistically reliable change. The outcome is deemed significant if a patient’s posttest score is within the functional range; a patient has reliably changed if the pretest-posttest change is larger than a chance fluctuation due to instrument measurement error. Various revisions of the Jacobson-Truax (JT) approach have been proposed [13–15], finding extensive application in comparing outcomes of groups of patients [16–18] as well as in ROM for individual patients [19]. Recently we evaluated the practicality of this approach as an indicator of institutional performance, using pretest and posttest scores on self-report measures. The JT approach was deemed a worthy addition to traditional performance indicators such as pretest-posttest ES or change in T-score (ΔT), as it illustrates these numerical values in a clinically meaningful manner with children and adolescents [20], and with adults with common mental disorders such as depression and anxiety disorders [9].

Application of JT to rating scales, such as the HoNOS, is less common than its application to self-report measures. The results appear of limited use when the JT approach is applied to HoNOS for the SMI population, as usually a very large proportion of patients is deemed unchanged. This may reflect the chronicity of SMI, where change – let alone(clinical) recovery or remission – is relatively uncommon within the time frame of one or two years. It may be caused by lack of responsiveness to change of the HoNOS, especially for patients with low pretest scores to begin with [21], but it may also be due to the stringency of JT criteria, particularly for reliable change [22]. In a paper published in 2005, Parabiaghi et al. [23] proposed for the HoNOS total score that a change of at least 8 points is required to deem a patient as statistically reliably changed. Such a change in score is substantial and infrequent in care provided to the majority of patients with SMI, but is also a stringent criterion when the HoNOS is applied to evaluate outpatient care for common mental disorders [21]. Other values for reliable change and alternative statistical approaches to arrive at performance indicators for use with the HoNOS have been proposed by Burgess et al. [24]. They discuss the merits of effect size (ES), reliable change index (RCI), and standard error of measurement (SEM), proposing various threshold values for these indicators to distinguish unchanged from changed patients (improved or deteriorated), varying in statistical uncertainty. Utilization of each threshold score yields three possible outcomes: no significant change, significant improvement, and significant deterioration.

In order to obtain an improved categorization for use with HoNOS data, in a more recent paper from 2014 Parabiaghi et al. [22] describe a revised approach to JT. This approach (JT_revised_) focuses more on outcome than on change, underlining the significance of slightly changed and unchanged subjects. Where JT distinguishes two classes of patients (dysfunctional and functional), Parabiaghi et al. propose three classes of severity for the HoNOS total score: mild (< 10), moderate (10–13), and severe (> 13). They also propose two levels of meaningful change: reliably changed (RCI 90%; at least 8 points) and minimally changed (at least 4 points change). Potentially, the method proposed by Parabiaghi et al. [22] is an improvement over the traditional JT approach: as it allows for a more comprehensive categorization of treatment results, it seems better suited to meet the demands of clinical reality.

In the present study, we compared several categorical approaches as clinical illustrations of ES and ΔT: classifications into three categories (improved, unchanged, and deteriorated) based on ES and RCI threshold values, dichotomous classifications (JT_RCI_ and JT_CS_), the more complex classification of JT into four categories (recovered, improved, unchanged, deteriorated, or JT_RCI&CS_), and the proposed revised JT of Parabiaghi et al. [22] into eight categories (JT_revised_). We evaluated which categorical method is most suitable to denote outcome for patients with SMI by comparing the ranking of institutions according to ES and/or ΔT with their ranking based on categorical outcomes. ES/ΔT was chosen as the reference method, as this outcome indicator is appropriate given the continuous nature of the data, and it is the most commonly used effect indicator to denote within-group effect size in treatment outcome research [8]. We therefore examined which of the categorical methods revealed the largest differences in outcome between mental health institutions, whether rankings based on continuous and categorical methods were concordant and evaluated the informative value of each method.

It is important to note that the aim of the present study was to compare performance indicators for their ability to assess differences in outcome of care among institutions. Variation in outcome between providers enables us to compare performance indicators. The aim was *not* to compare the performance of the participating institutions per se. Case mix differences and differences in completeness of the data among institutions preclude firm conclusions regarding their comparative performance. We consequently choose to anonymize institutions. The reader should take note of the fact that ranking of institutes does not necessarily reflect an order in the quality of care provided; it is merely a reflection of differences in outcome, which may well be due to case mix differences or other factors affecting outcome, such as timing of assessments, proficiency in use of the HoNOS, etc.

## Methods

### Design and participants

This is an observational study, using data from real-life patients in everyday clinical practice. Data were collected from 10 integrated mental health institutions in the Netherlands and pertain to first-year-of-care episodes completed in 2013 and 2014. Participating institutions offer a mix of inpatient and intensive outpatient treatments, day-clinic treatment, and what is known as (Flexible) Assertive Community Treatment ((F)ACT). Patients receiving short-term crisis intervention were excluded. Data were collected as part of the treatment and anonymized before analysis. Patients were informed about use of the data for routine outcome monitoring [2], and Dutch law allows use of these anonymized ROM data for research [25]. The Central Committee on Medical Research (CCMO) approved the use of anonymized data. The study included data from *N* = 3189 patients. Institutions contributed between 199 and 505 cases (*M* = 318,9; *SD* = 106.7; see Table 1). Institutions are given a number that represents their position in the rank order from worst to best outcome according to ES and ΔT (both rankings are almost identical; where ties occur in one indicator, ranking of tied institutions is based on the other indicator).

**Table 1:** Number of patients, gender, age, pretest-posttest response rate, and length of treatment (days) per institution

Institution		1	2	3	4	5	6	7	8	9	10	Total
Included	N	1056	771	968	4754	2061	9221	2177	2076	1304	589	16,771
Assessed	N	265	192	215	290	336	342	505	438^a^	407	199	3189
	%	8.3	6.0	6.7	9.1	10.5	10.7	15.8	13.7	12.8	6.2	100.0
Response	%	25.1	24.9	22.2	6.1	16.3	33.7	23.2	21.1	31.2	33.8	19.1
Length of	M	337.7	328.9	326.8	284.1	312.4	314.0	288.6	294.8	225.6	329.9	297.9
treatment	SD	51.8	57.4	54.6	99.2	73.2	80.5	91.8	85.8	138.9	41.8	93.5
Males	N	164	121	130	238	170	227	282	155	248	123	1858
	%	61.9	63.0	60.5	82.1	50.6	66.4	55.8	35.4	60.9	61.8	58.3
Age	M	40.0	40.2	42.7	38.8	42.5	37.8	42.7	40.6	39.0	42.6	40.7
	SD	12.4	12.8	11.5	10.6	11.6	13.0	12.3	11.9	12.4	13.8	12.3
HoNOS	M	9.7	14.0	13.6	14.9	13.3	11.2	11.5	12.3	13.3	10.3	12.4
pretest	SD	5.4	3.0	2.0	7.5	6.0	5.8	6.1	6.4	7.2	6.1	6.5
HoNOS	M	9.4	12.1	12,8	13.8	11.5	9.5	9.6	9.8	10.5	7.9	10.5
posttest	SD	6.4	6.8	7.0	6.9	5.9	5.8	6.0	6.2	6.8	5.8	6.5

^a^For 112 (12.1%) of 438 patients of institution 8 no information on gender was provided

### Instrument

#### Health of nation outcome scales (HoNOS)

The Health of Nation Outcome Scales (HoNOS) was developed in 1993 by the Research Unit of the Royal College of Psychiatrists to evaluate clinical treatment outcome [5]. The HoNOS is a rating scale, to be administered by a trained practitioner or research assistant. The instrument is short and easy to complete, and it was designed for routine clinical work. Its use is widespread, as it is the prime outcome measure for mental health care in the UK [6], Australia [26], and New Zealand [27]. In the Netherlands, use of the HoNOS is limited to patients with SMI, who receive “integrated care , i.e. support living, work, and social relations in addition to psychiatric treatment” [25]. It consists of 12 items that cover clinical problems and social functioning. Each item is evaluated on a 5-point (0–4) Likert scale, resulting in a total score ranging from 0 to 48. Response options vary for each item and are anchored with a comprehensive description. Several studies have evaluated the HoNOS and found support for its reliability, validity, and sensitivity to change [25, 27, 28].

### Methods for rendering treatment outcome

#### Continuous indicators (ES and ΔΤ)

A popular estimate of treatment outcome is the within-group effect size estimator ES, denoting the size of the pretest-posttest change in standardized units [8]. It provides a clear indication of what has been achieved in treatment [29], and is calculated as the difference of pretest and posttest scores divided by the pretest standard deviation of the instrument for patients.

ΔT is an outcome indicator similar to ES, but based on the difference between pretest and posttest scores transformed to standardized T-scores [30] with a normal distribution of scores. Raw HoNOS scores are asymmetrical and skewed to the right, which implies that intervals in the lower scale range are not equal to intervals in the higher range. Normalization turns the HoNOS into a true interval scale, a measurement level required for subtraction [31]. T-scores have a standard deviation of 10, therefore ΔT is similar to ES but 10 times larger. For the present study, categorical indicators based on raw scores were compared to ES, and categorical indicators based on T-scores were compared to ΔT.

#### Categorical indicators based on ES, RCI and RCI threshold values

Burgess, Pirkis and Coombs [24] propose various threshold values for ES, RCI, and SEM when using raw HoNOS scores to classify patients as unchanged, improved or deteriorated. Significantly changed are patients with a change of at least ES_medium_ = 4 or ES_large_ = 6, four RCI thresholds with different confidence levels (RCI_95_ = 10, RCI_90_ = 9; RCI_80_ = 7 and RCI_67_ = 5), and a single threshold value based on SEM proposed by McHorney and Tarlov [32]. SEM is calculated by multiplying the standard deviation by the square root of 1 minus the reliability coefficient. For the HoNOS when used with inpatients Burgess et al. [24] propose SEM = 5.

#### Categorical indicators based on the JT approach

##### Traditional Jacobson-Truax (JT_CS_, JT_RCI_ and JT_RCI&CS_)

JT is a widely accepted approach to denote clinically significant change in patients and to identify meaningful individual improvement [16]. Based on the criteria described in the Introduction, JT yields three indicators: (a) clinical significance composed of a cut-off point where “the patient moves outside the dysfunctional population or within functional population” (JT_CS_); (b) reliable change index, which indicates whether the change that occurred was statistically significant (JT_RCI_); and (c) the combination of these two (JT_RCI&CS_), which categorizes outcome of treatment into “deteriorated”, “unchanged”, “improved” or “recovered” [11]. We applied the traditional JT approach to raw scores (RCI = 8, CS = 5) [23] and to transformed T-scores (RCI = 5 and CS = 42.5) [9, 20].

### Revised JT model

Parabiaghi et al. [22] proposed a revised model of JT to denote meaningful clinical outcome for patients with SMI, using two change and two endstate threshold values. First, they proposed three levels of change: reliable change (RCI: change ≥8), minimal change (based on the SEM: change ≥4), or no change or stability (change < 4). They also proposed distinguishing three levels of severity: mild (HoNOS score < 10), moderate (score = 10–13), and severe (score > 13). All in all, this combination of two change criteria and two severity cut-offs leads to a complex “research model” comprising 23 outcome categories (see Parabiaghi et al. [22]; Fig. 1, p. 299). For clinical use they propose a simplified version using a single change criterion (at least 4 points change, the more lenient criterion for a minimally detectable change based on SEM; the chosen value of 4 is based on data of Italian and Dutch patients) and the two CS values (10 and 13). Combination of three possible outcomes according to SEM-based minimally detectable change (improved, unchanged, or worsened) and three severity levels (mild, moderate, and severe) results in a categorization into nine groups (see Parabiaghi et al. [22]; Fig. 2, p. 300). Stable patients are categorized into three levels: “stability in mild illness”, “stability in moderate illness”, and “stability within severe illness”, utilizing the cut-off values of 10 and 13 on the means of their pretest and posttest HoNOS scores. Based on the posttest HoNOS score, three improved groups are distinguished: “improved to mild illness”, “improved to moderate illness”, and “improved within severe illness”. As worsening to a mild level is a relatively rare event, those who showed significant worsening from pretest to posttest were allocated to only two categories: “worsening to mild or moderate illness” and “worsening to severe illness”, using the cut-off value of 13 to distinguish between the two groups [22]. Hence the simplified JT_revised_ model results in eight outcome categories (see Table 5).

### Statistical analysis

Outcomes between institutions were compared and the ability of the various indicators to distinguish between them was investigated. A repeated-measures ANOVA for a 2 (time) × 10 (institution) design was conducted on transformed T-scores to test for main effects of time and institution as well as for their interaction to compare outcome slopes of institutions over time. Post hoc pairwise comparisons were conducted to assess which institutions differed from each other. For the categorical outcomes we assessed the differences in proportions with chi-squared tests. Ranking of institutes according to each outcome indicator is presented and compared to ranking according to ES or ΔT (the reference indicator for raw scores or T-scores, respectively). Concordance between rankings is assessed with the Spearman Rank correlation coefficient.

## Results

The initial dataset comprised 16,771 patients who received treatment; 8402 (50.1%) were assessed at pretest and for 38.0% of these posttest data were available, yielding a final sample of 3189 patients with complete pretest and posttest data and an overall response rate of 19.1%. Table 1 presents background information on the participating patients. The duration of care episodes ranged from 30 to 446 days (M = 297.8; SD = 93.4), with no significant differences between institutions. Pretest selection, posttest attrition and overall response rates (the proportion of care episodes with complete pretest and posttest data) varied considerably between institutions (range: 6.1–33.8%).

There were statistically significant, albeit small, differences between institutions in mean age and gender; overall 58.3% of patients were males and gender was unevenly distributed among the 10 institutions (χ^2^(9) = 103.44; *p* < .001), with Institution 4 treating more males (82.1% vs. 58.3% for the total population). Participants’ age ranged from 17 to 84 years (M = 40.7; SD = 12.3) and varied among institutions (F(9) = 7.34; *p* < .001; η^2^ = .02), with Institutions 4, 6, and 9 treating somewhat younger patients. The mean pretest score on the HoNOS differed significantly between institutions (F(9) = 18.04; p < .001; η^2^ = .05), with Institutions 1, 6, and 10 having lower scores (i.e. less impairment in function) than the others according to Bonferroni corrected pairwise comparisons.

There were large differences in diagnostic composition of the case mix among institutions. Table 2 presents this diagnostic information. The largest group among the diagnoses is psychotic disorders (47.3%), followed by mood/anxiety/somatoform disorders (17.9%) and personality disorders (11.4%). The smallest groups are pervasive developmental disorders (10.5%), substance abuse (5.0%), and bipolar disorder (4.9%). Patient composition differs significantly among institutions (χ^2^(54) = 1583.09; *p* < .001), with Institution 4 treating more patients with substance-related disorders (36.9%) and fewer psychotic disorders (13.4%), Institution 5 treating more patients with mood/anxiety/somatoform disorders (36.0%) and fewer personality disorders (18.8%), Institution 6 treating more patients with pervasive developmental disorders (44.7%), and Institutions 8, 5, and 1 treating more personality disorders (19.4, 18.8 and 17.7%, respectively).

**Table 2:** Overview of the case mix composition regarding main psychiatric diagnosis per institution

Institution		1	2	3	4	5	6	7	8	9	10	Total
Psych. Dis.	N	132	128	127	39	108	108	282	195	264	125	1508
	%	49.8	66.7	59.1	13.4	32.1	31.6	55.8	44.5	64.9	62.8	47.3
MAS	N	27	23	25	61	121	29	110	80	64	31	571
	%	10.2	12.0	11.6	21.0	36.0	8.5	21.8	18.3	15.7	15.6	17.9
Pers. Dis.	N	47	11	21	38	63	28	37	85	21	12	363
	%	17.7	5.7	9.8	13.1	18.8	8.2	7.3	19.4	5.2	6.0	11.4
Perv. DD	N	32	20	14	36	17	153	29	15	11	9	336
	%	12.1	10.4	6.5	12.4	5.1	44.7	5.7	3.4	2.7	4.5	10.5
Bipolar Dis.	N	18	5	7	4	14	17	12	23	38	18	156
	%	6.8	2.6	3.3	1.4	4.2	5.0	2.4	5.3	9.3	9.0	4.9
Substance	N	4	2	17	107	6	4	5	12	0	1	158
	%	1.5	1.0	7.9	36.9	1.8	1.2	1.0	2.7	0.0	0.5	5.0
Other	N	5	3	4	5	7	3	30	28	9	3	97
	%	1.9	1.6	1.9	1.7	2.1	0.9	5.9	6.4	2.2	1.5	3.0

*Psych. Dis.* Psychotic Disorders, *MAS* Mood, anxiety, and somatoform disorders, *Pers. Dis.* Personality Disorders, *Perv. DD* Pervasive Developmental Disorders, *Bipolar Dis.* Bipolar Disorders, *Substance* Substance Abuse

Difference between pretest-posttest change on HoNOS T-scores among institutions was analyzed in a 2 (time) × 10 (institution) repeated-measures ANOVA. This revealed statistically significant main effects of time (F(1) = 233.4; *p* < .001; η^2^ = .068) and institution (F(9) = 23.6; *p* < .001; η^2^ = .063), which reflects a difference over time as well as between institutions regardless of time. More importantly, there was a significant interaction effect (time x institution) revealing a difference in outcome slope between health institutions over time (F(9,3179) = 3.33; *p* < .001; η^2^ = .009). Pairwise comparisons of institutions (with Bonferroni correction) revealed that Institutions 5 to 10 reported larger pretest-posttest differences than Institutions 1 to 4.

Ranking of institutions was based on ES and ΔT. Hence, institutions with a higher rank number have a larger ES than those with a low rank number, as Table 3 shows. This table also presents results using threshold values for ES, SEM, and JT_RCI90_. All categorizations reveal significant differences among institutions (all *p* < .001). The proportions of reliably changed patients from Table 3 using the RCI threshold of at least 8 points as proposed by Parabiaghi et al. [23] varied among institutions (χ^2^(9) = 58.1; p < .001), as did the proportions of patients with a posttest score < 5, denoting a clinically significant change (χ^2^(9) = 42.8; p < .001). Finally, combining both indices in four outcome categories also reveals differences among institutions (χ^2^(27) = 111.4; *p* < .001). Institutions with a higher rank number had more recovered (= Institution 9: 17.4% vs. = Institution 1: 4.9%) and fewer deteriorated patients (= Institution 9: 10.6% vs. Institution 6: = 3.8%). The results indicate that 11.2% (*n* = 356) of patients had recovered, 6.1% (*n* = 196) had improved, 75.9% (*n* = 2421) remained unchanged, and 6.7% (*n* = 215) had deteriorated. The large proportion of unchanged patients results from the stringent RCI criterion of at least 8 points change. The ranking of institutes diverges considerably among indicators, and most indicators have no statistically significant association with ES, except for the improved and reliable change (JT_RCI_) indicators, which correspond best with ES. All in all, most of the indicators proposed by Burgess et al. [24] and JT_RCI&CS_ based on raw scores are insufficiently concordant with ES.

**Table 3:** Effect Size (ES) and percentage of patients in outcome categories based on raw HoNOS scores and classification according to various ES_medium_, SEM, and JT_RCI-90_ threshold values, and according to JT_RCI95_, JT_CS_, and JT_RCI&CS_

Institution		1	2	3	4	5	6	7	8	9	10	Total	Rank order^a^	Rho
Continuous:	ES	0.06	0.18	0.19	0.24	0.29	0.29	0.32	0.38	0.38	0.39	0.29	1 2 3 4 5/6 7 8/9 10	
Categorical:														
ES_medium_ ≤ −4	deteriorated	20.0	19.3	20.5	20.7	15.2	11.4	17.8	14.8	20.9	14.1	17.3	9 4 3 1 2 7 5 8 10 6	.35
-4 < ES_medium_ < 4	unchanged	56.2	48.4	43.7	40.0	49.7	57.6	45.0	42.7	34.4	46.7	45.9	6 1 5 2 10 7 3 8 4 9	.38
ES_medium_ ≥ 4	improved	23.8	32.3	35.8	39.3	35.1	31.0	37.2	42.5	44.7	39.2	36.8	1 5 2 8 4 3 6 9 10 7	.71*
SEM ≤ −5	deteriorated	15.5	13.0	15.3	17.6	12.5	9.1	14.5	11.0	16.2	11.1	13.5	4 9 1 3 7 2 5 10 8 6	.35
-5 < SEM < 5	unchanged	63.4	60.4	56.3	49.3	57.1	68.1	53.7	52.7	45.9	54.8	55.5	6 1 2 5 3 10 7 8 4 9	.55
SEM ≥ 5	improved	21.1	26.6	28.4	33.1	30.4	22.8	31.9	36.3	37.8	34.2	30.9	1 6 2 3 5 7 4 10 8 9	.79**
JT_RCI90_ ≤ −9	deteriorated	8.3	4.2	7.0	9.3	3.6	2.3	5.1	3.9	7.6	2.5	5.4	4 1 9 3 7 2 8 5 10 6	.43
9 < JT_RCI90_ < 9	unchanged	85.3	82.8	80.0	71.4	87.5	88.0	82.2	79.2	69.0	83.9	80.6	6 5 1 10 2 7 3 8 4 9	.24
JT_RCI90_ ≥ 9	improved	6.4	13.0	13.0	19.3	8.9	9.6	12.7	16.9	23.3	13.6	14.1	1 5 6 7 2/3 10 8 4 9	.51
JT_RCI_ (change ≥8)	reliable change	9.4	14.6	14.9	19.7	11.3	14.3	17.4	21.7	27.0	15.6	17.3	1 5 6 2 3 10 7 4 8 9	.64*
JT_CS_ (post < 5)	clinical change	13.6	9.9	7.0	7.2	8.6	14.9	16.0	15.5	15.2	21.1	13.3	3 4 5 2 10 1 6 8 7 9	.56
JT_RCI&CS_	deteriorated	9.1	5.2	8.4	10.3	3.9	3.8	5.9	5.7	10.6	4.5	6.7	9 4 1 3 7 8 2 10 5 6	.14
	unchanged	81.5	80.2	76.7	70.0	84.8	81.9	76.6	72.6	62.4	79.9	75.9	5 6 1 2 10 3 7 8 4 9	.39
	improved	4.5	4.7	3.3	4.1	3.0	6.1	6.3	8.7	9.6	8.0	6.1	5 3 4 1 2 6 7 10 8 9	.75*
	recovered	4.9	9.9	11.6	15.5	8.3	8.2	11.1	13.0	17.4	7.5	11.2	1 10 6 5 2 7 3 8 4 9	.24

$$ ES=\frac{M_{pretest}-{M}_{posttest}}{SD_{pretest}}; $$ $$ SEM= SD\times \sqrt{1-{r}_{ii}} $$; $$ {JT}_{RCI}=\frac{M_{pretest}-{M}_{posttest}}{SEM} $$; $$ {JT}_{CS}=\frac{SD_{pre}\ast {M}_{post}+{SD}_{post}\ast {M}_{pre}}{SD_{pre}+{SD}_{post}} $$

ES_medium_ implies a change of at least 4 points; SEM implies 5 points change or more; JT_RCI90_ implies 9 points change; JT_RCI90_, JT_CS_, and JT_RCI&CS_ imply 8 points change and transgression of a score of 5 from pretest to posttest. Rho is Spearman rank correlation coefficient between the ranking based on an indicator and ranking based on ES (*p < .05; **p < .01)

^a^Ranked from the worst (1) to the best (10) outcome according to the indicator; categories “unchanged” and “deteriorated” have a reversed rank order: the fewer the patients the better the outcome

Table 4 presents the results when we convert the HoNOS scores to T-scores. Again, institutions with a high rank number performed better (ΔT = range 3.3–4.3) than those with a lower rank number (ΔT range 0.9–3.0). Using the threshold of a change ΔT >  5 [9, 20], the proportions of reliably changed patients differed significantly among institutions (χ^2^(9) = 29.8; *p* < .001), as did the proportions of patients transgressing the threshold of CS = 42.5 (pretest ≥42.5; posttest < 42.5), denoting clinically significant change (χ^2^(9) = 30.4; *p* < .001). Combining the two indices into JT_RCI&CS_ with four categories also reveals significant differences among institutions (χ^2^(27) = 76.1; *p* < .001). Furthermore, with the traditional JT_RCI&CS_ method applied to T-scores, patients got more evenly distributed over the outcome categories: in total 18.8% (*n* = 598) of patients were considered recovered, 22.2% (*n* = 709) had improved, 40.0% (*n* = 1277) remained unchanged, and 19.0% (*n* = 605) had deteriorated. Institutions with a higher rank have more recovered patients (Institution 10: 24.6% vs. = Institution 1: 12.5%) and fewer deteriorated patients (Institution 10: 17.1% vs. = Institution 1: 23.4%). The Rho correlation coefficients indicate that the rankings based on ΔT scores (in Table 4) are more concordant than rankings based on raw HoNOS difference scores (ES in Table 3), with JT_RCI&CS_ recovery having the highest concordance with ΔT, followed by the category of unchanged patients. However, lack of concordance is also noteworthy. Institution 9, for instance, has the second-highest ranking based on ΔT, but also the third-largest proportion of deteriorated patients (based on JT_RCI&CS_; see Table 4).

**Table 4:** Mean ΔT and percentage of patients in outcome categories based on T-scores according to the JT approach

Institution		1	2	3	4	5	6	7	8	9	10	Total	Rank order^a^	Rho
Continuous:	Mean ΔT	0.9	2.0	2.0	2.6	2.8	3.0	3.3	4.1	4.3	4.3	3.1	1 2/3 4 5 6 7 8 9/10	
Categorical:														
JT_RCI-95_ (> 5)	improved	33.6	34.4	37.7	40.0	37.5	36.5	43.0	46.8	46.9	45.7	41.0	1 2 6 5 3 4 7 10 8 9	.86**
JT_CS_ (42.5)	changed	14.0	14.1	19.1	14.8	17.6	20.5	23.6	23.1	23.8	25.6	20.2	1 2 4 5 3 6 8 7 9 10	.95**
JT_RCI&CS_	deteriorated	23.4	18.2	21.4	22.1	16.1	14.3	20.2	16.0	21.9	17.1	19.0	1 4 9 3 7 2 10 5 8 6	.38
	unchanged	43.0	47.4	40.9	37.9	46.4	49.1	36.8	37.2	31.2	37.2	40.0	6 2 5 1 3 4 8/10 7 9	.65*
	improved	21.1	21.4	19.1	26.9	22.0	18.7	21.4	24.9	23.6	21.1	22.2	6 3 1 10 2/7 5 9 8 4	.26
	recovered	12.5	13.0	18.6	13.1	15.5	17.8	21.6	21.9	23.3	24.6	18.8	1 2 4 5 6 3 7 8 9 10	.93**

^a^Institutions are rank ordered according to increased performance (the higher the rank number the more improved or recovered patients); categories “unchanged” and “deteriorated” have a reversed rank order (fewer patients means a higher rank and a better outcome)

*p < .05; **p < .01

The results of Table 5 show the categorization according to the revised JT proposed by Parabiaghi et al. (2014). A significant difference among institutions in these categories is found, with higher rates of patients in the “mild illness” and “improvement to mild illness” categories and lower rates of “stability in severe illness” or “worsening in/to severe illness” among institutions with a high ranking (χ^2^(63) = 230.9; *p* < .001). Correspondence between ranking of institutes according to ES and the JT_revised_ categorization is low, except for the category “improvement to mild illness”.

**Table 5:** Percentage of subjects classified into 8 outcome categories based on raw scores according to the revised JT approach of Parabiaghi et al. [22]

institution	1	2	3	4	5	6	7	8	9	10	Total	Rank order^a^	Rho
Mild illness	40.4	17.7	21.4	15.2	22.9	36.3	29.3	24.9	21.1	34.2	26.4	4 2 9 3 5 8 7 10 6 1	.13
Improvement to mild illness	18.5	17.7	22.3	18.3	20.5	22.8	26.9	28.3	31.2	32.7	24.6	2 4 1 5 3 6 7 8 9 10	.92**
Improvement to moderate illness	3.8	6.8	7.0	11.4	7.7	4.4	6.9	8.2	7.1	4.5	6.9	1 6 10 2 7 3 9 5 8 4	.23
Improvement within severe illness	1.5	7.8	6.5	9.7	6.8	3.8	3.4	5.9	6.4	2.0	5.3	1 10 7 6 8 9 3 5 2 4	−.29
Stability in moderate illness	12.1	12.0	8.4	9.3	10.4	11.4	9.1	7.8	5.9	8.0	9.2		
Worsening to moderate illness	4.9	5.2	3.3	2.8	2.7	2.0	4.0	3.9	4.4	3.0	3.6	2 1 9 7 8 3 10 4 5 6	.26
Stability in severe illness	7.2	20.3	15.8	17.9	18.8	11.7	8.9	11.4	10.3	8.0	12.5		
Worsening in/to severe illness	11.7	12.5	15.3	15.5	10.1	7.6	11.5	9.6	13.5	7.5	11.4	4 3 9 2 1 7 5 8 6 10	.52

Improvement based on SEM: pre-to-posttest change ≤4

Mild illness: post < 10; moderate illness: post = 10–13; severe illness post > 13

^a^Rank order is not provided for stability categories; rank order of worsening is reversed

**p < .01

## Discussion

In the present study, we compared various categorical indicators on their usefulness to illustrate differences between institutions regarding treatment outcome. The primary aim of the study was to test the suitability of various categorical methods to denote treatment outcome in mental health care for patients with SMI using the HoNOS as assessment instrument. We were fortunate to find differences in outcomes between institutions and could use their data to evaluate various methods to delineate outcome. We also assessed the suitability of a number of methods to compare institutions. The results revealed differences in ranking institutions between the two continuous indicators (ES and ΔT) and the categorical indicators (SEM, JT_RCI_, JT_CS_, JT_RCI&CS_, JT_revised_). Indicators based on categorical outcomes yielded quite divergent rankings; the categories of the traditional JT approach were most concordant with the continuous outcome indicators ES and ΔT, particularly JT_RCI_ and JT_CS_ based on T-scores.

The traditional JT approach (JT_RCI&CS_) with four categories is applied frequently in practice and provides useful information on patients’ condition after treatment [9, 11, 16]. However, as an outcome indicator for aggregated data it has some serious drawbacks. As the indicator classifies patients into four categories, it is impossible to rank health institutions consistently: ranking according to proportion of recovered patients yields a different order than ranking according to proportion of reliably changed patients, and so forth. A possible solution would be to collapse the four categories into two, in order to get a ranking based on less complex information, but this reduces information value and statistical power. Fedorov, Mannino and Zhang [33] calculated that dichotomizing information leads to a substantial loss of statistical power (at least 36% reduction when data are made binary and 19% when data are converted to three categories). These percentages are based on optimal cut-off points. In practice, the loss of statistical power may be greater. Indeed, Markon, Chmielewski and Miller [34] showed that a sample needed to be twice as large when moving from a continuous to a dichotomous outcome. Statistical power can be increased by adding more categories, but this reintroduces the complexity of interpreting the outcome data.

Another drawback of the JT_RCI&CS_ method is that it will result in a large proportion of “unchanged” patients if a stringent criterion for RCI ≥ 8 is applied to raw HoNOS scores. Such a large category provides little information and is hard to interpret, as we are unsure whether to regard this outcome as disappointing or as successful stabilization (this of course also depends on the goal of treatment or care). Using various alternative cut-off values for deterioration or improvement, as proposed by Burgess et al. [24], does not lead to a categorization highly concordant with ES. The present results show that applying the JT categorization after raw scores have been converted into transformed T-scores yields a more even distribution of patients over the outcome categories. Moreover, ranking of institutes according to proportion of recovered patients based on transformed T-scores is more concordant with outcome according to ΔT than the ranking using raw scores. We therefore recommend using transformed T-scores with the proposed cut-off values RCI >  5 and CS = 42.5 – corresponding to RCI > 2 to RCI > 4 (depending on the position on the scale) and CS = 8 in raw score on the HoNOS – as the most suitable approach to convey differences in performance between institutions, given that this indicator is methodologically sound as it uses data that have been transformed into a normal distribution.

Parabiaghi et al. [22] evaluated a more refined approach for meaningful change and outcome. We examined this approach and compared it with the traditional JT approach, to investigate how these categorical methods compare in their convergence with the continuous method and how they compare in denoting outcome in a meaningful way. The results indicate that the proposed revision may have advantages over the traditional JT approach, as it provides a quite meticulous and clinically meaningful way to denote clinical status and outcome of care for individual patients with SMI. JT_revised_ may thus be more informative for clinicians when monitoring progress and choosing the most appropriate course of treatment as compared to the traditional JT approach. Further validation of JT_revised_ is needed to justify use of its more refined outcome categories. It should also be noted that the threshold value for change based on SEM (change ≥4 is deemed meaningful) needs validation, as it is far more lenient than the RCI90 ≥ 9 based on the formulas proposed by Jacobson and Truax and the reliability of the HoNOS may not justify the chosen low-threshold value. Future research, for instance directly comparing the predictive validity of the categorization according to the traditional JT approach and the JT_revised_ in terms of further course of treatment, will reveal which approach best predicts need for care after the first year. However, the Parabiaghi approach is deemed too complex for research on groups of patients or for use as a performance indicator comparing aggregated outcomes of institutions: with eight categories it is not considered a practical or more appropriate alternative to the simpler traditional JT_RCI&CS_ with four categories.

A strength of the present study is its use of real-life data, collected in everyday clinical practice. The study also uses a considerably large data set, in number of both institutions and patients per institution, boosting confidence in the generalizability of the findings for clinical practice in the Netherlands and bringing about ample statistical power to find differences among methods to denote outcome. Indeed, substantial variation in outcome was found among institutions, offering a realistic test of the usefulness of various approaches to denote outcome of patients in care for SMI.

A limitation of the study is that only data from the first year of care were analyzed. Patients with SMI typically stay in care for a longer period. Their change in subsequent years of care is likely to be substantially smaller, as may also be the case for outcome variation between institutions. It should be noted that the substantial differences between institutions in case mix composition for demographics and clinical features of patients, as well as differences in completeness of provided data, imply that outcomes of institutions are potentially confounded by these pretest differences. For example, institutions’ patient populations vary in pretest severity, a variable strongly associated with posttest scores and gain scores; this implies that the level of pretest severity is also associated with categorical outcomes. Higher average pretest levels leave more room for reliable improvement, lower pretest levels leave less room but make achieving recovery status more likely. In addition, case mix composition between institutions also differed in ratio of inpatients to community patients. This underscores the need for case mix correction when comparing institutional performance. We reanalyzed the data after case mix correction for several variables that appeared associated with outcome (pretest severity, age, and bipolar disorder). This case mix model explained 23% of outcome variation (predominantly by pretest HoNOS scores). Correction did influence average outcome of institutions, but overall the ranking of institutes remained the same. However, differences between institutions diminished somewhat, and with this smaller contrast between institutions the rankings of the various approaches were more diverse. Consequently, the concordance between approaches was also more varied. As a further limitation of the study, response rates for institutions ranged from 6.1 to 33.8%, compromising the representativeness of the data for the institutions. Hence, the present results do not necessarily reflect differences in quality of care between institutions and should be examined cautiously, also bearing in mind that comparing institutions was not our aim. Moreover, the overall response rate limits the generalizability of the study findings, as we do not know whether outcome data are missing systematically.

The HoNOS total score may be considered too small a basis to evaluate the outcome of an individual patient or appraise the overall performance of mental health institutions. Use of the HoNOS is widespread, not only for outcome monitoring but also to assign patients to clusters based on their treatment needs. Large datasets have thus become available to evaluate the psychometric quality of the instrument, and some negative findings have emerged. For instance, the HoNOS appears not to be associated with need-for-care as operationalized by costs of treatment in a large British cohort of 1343 patients with common mental health problems [35]. For this patient group, the sensitivity to change in severity of psychopathology of the HoNOS appears to be limited as only three items (7, 8, and 9) seem relevant and appropriate [21]. The utility of the HoNOS for clustering patients into groups of various need levels has been questioned as well [36]. Finally, the factorial structure of the HoNOS has been criticized: the HoNOS does not appear to be unidimensional, which casts doubt on the validity of calculating a total score. Various multidimensional factorial models have been proposed, but none appears to have sufficient fit to be deemed good over the full range of psychiatric disorders [37]. Further development of measurement instruments for appropriate outcome domains (assessing severity of symptomatology, functioning, and personal recovery) is therefore needed, and several such projects are currently underway, internationally as well as in the Netherlands. Finally, the present study lacks an external criterion to validate the various methods to denote outcome. Additional information on patients’ posttreatment functioning is needed, such as continued use of mental health care after the first year of treatment or long-term follow-up data (e.g. several years after treatment has ended).

## Conclusions

Methods based on continuous variables – ES on raw scores or ΔT based on transformed T-scores – are the most convenient choice for research or for comparing institutions, subdivisions or teams: they have the best statistical power and allow for a straightforward ranking of institutions. Based on this study, we conclude that the use of categorical approaches is complicated as it matters importantly which outcome category is considered for ranking institutions on their performance. However, information from categorical approaches is of *supplemental* value, as this illustrates what differences in rank order mean in clinically relevant terms and reveals what has been accomplished in clinically meaningful terms. We recommend the traditional JT approach as a good choice among the categorical indicators. The revision by Parabiaghi et al. [22] provides more detailed information, but eight outcome categories may be too complex for a practical comparison of institutions.

## Abbreviations

CS: Clinical Significant change
ES: Effect Size
HoNOS: Health of Nation Outcome Scale
JT: Jacobson-Truax
RCI: Reliable Change Index
SEM: Standard Error of Measurement
ΔT: Difference T-score from pretest to posttest
